# Human Papillomavirus (HPV) Vaccination and Adolescent Girls' Knowledge and Sexuality in Western Uganda: A Comparative Cross-Sectional Study

**DOI:** 10.1371/journal.pone.0137094

**Published:** 2015-09-01

**Authors:** Andrew Kampikaho Turiho, Wilson Winston Muhwezi, Elialilia Sarikiaeli Okello, Nazarius Mbona Tumwesigye, Cecil Banura, Anne Ruhweza Katahoire

**Affiliations:** 1 Department of Psychiatry, School of Medicine, Makerere University, Kampala, Uganda; 2 School of Public Health, Makerere University, Kampala, Uganda; 3 Child Health and Development Center, Makerere University, Kampala, Uganda; State University of Maringá/Universidade Estadual de Maringá, BRAZIL

## Abstract

The purpose of the study was to investigate the influence of human papillomavirus (HPV) vaccination on adolescent girls’ knowledge of HPV and HPV vaccine, perception of sexual risk and intentions for sexual debut. This cross-sectional comparative study was conducted in Ibanda and Mbarara districts. Data was collected using a standardized self-administered questionnaire and analyzed using the Statistical Package for the Social Sciences computer software. Univariate, bivariate, and logistic regression analyses were conducted with significance level set at *p* < .05. Results showed that HPV vaccination was associated with being knowledgeable (Crude OR: 5.26, CI: 2.32–11.93; *p* = 0.000). Vaccination against HPV did not predict perception of sexual risk. Knowledge was low (only 87/385 or 22.6% of vaccinated girls were knowledgeable), but predicted perception of a high sexual risk (Adjusted OR: 3.12, CI: 1.37–3.63; *p* = 0.008). HPV vaccination, knowledge and perceived sexual risk did not predict sexual behaviour intentions. High parental communication was associated with adolescent attitudes that support postponement of sexual debut in both bivariate and multiple regression analyses. In conclusion, findings of this study suggest that HPV vaccination is not likely to encourage adolescent sexual activity. Influence of knowledge on sexual behaviour intentions was not definitively explained. Prospective cohort studies were proposed to address the emerging questions.

## Introduction

Globally, about 500,000 new cases of cervical cancer and about 274,000 deaths due to the disease occur annually. More than 80% of these deaths occur in developing countries, where cervical cancer is the leading cause of cancer deaths among adult women. This is projected to increase to 90% by 2020 [1]. The human papillomavirus (HPV), which causes most of the cervical cancer is primarily sexually transmitted [2]. Two prophylactic HPV vaccines, Cervarix (bivalent) and Gardasil (quadrivalent), have been proven to be 90% effective in safely preventing HPV 16 and 18 infections, which together account for about 70% of cases worldwide [3]. The HPV vaccine mainly targets young adolescent girls and is most effective if given to HPV-free girls. Globally, studies indicate an overall positive attitude toward vaccination of young adolescents against the HPV [2–7]. However, in targeting young adolescent girls, which is a group not usually vaccinated, the HPV vaccine tends to arouse anxiety especially among parents. Several studies report a few people that oppose vaccination of young adolescents against sexually transmitted infections (STIs) in general or the HPV in particular out of fear that it could have inadvertent negative influence on their sexual behavior [5, 8–17]. This attitude is informed by two main hypotheses. It is suspected that the HPV vaccination may ignite the girls’ sexual curiosity leading to early sexual activity since it could foster the false belief that the vaccine protects against all STIs. Secondly, the availability of the HPV vaccine for young girls and the parental consent that they get vaccinated against the HPV may be perceived by the girls as implicit societal and parental approvals to be sexually active [18]. On the contrary, it is hypothesized that the vaccine against STIs could provide a key opportunity for increasing young people’s awareness of their risk to acquiring these infections when they become sexually active, and the need for prevention [13, 19]. The essence of this hypothesis is that if HPV vaccination is packaged together with clear messages about the HPV, purpose and limits of the vaccine it would enlighten adolescents about the risks of unsafe sexual behaviours and the need for preventive measures against STIs like postponement of sexual debut (PSD).

From 2008 to 2011, cohorts of adolescent girls in primary schools in Ibanda and Nakasongola districts in Uganda were vaccinated against the HPV using the bivalent vaccine (Cervarix, by GlaxoSmithKline of Britain). This was part of a demonstration project by the Government of Uganda and the Program for Appropriate Technology for Health (PATH) to evaluate different HPV vaccine delivery strategies. A school-based HPV vaccine delivery strategy was adopted in Ibanda targeting girls enrolled in primary grade five (P5). In Nakasongola, the HPV vaccine was delivered during the routine Child Days Plus (CDP) program, targeting girls of at least 10 years. Each eligible girl was to receive three doses of the vaccine administered in month 1, month 2 and month 6 [4]. Prior to commencement of vaccination, the girls and communities in general underwent health education for weeks. The education was conducted through various channels including; IEC materials (posters, leaflets for parents and girls, fact book for teachers and handbook for health workers), radio (phone-in talk programs, spot messages and announcements), and mobile film van screening a 30-minute documentary about cervical cancer. Other channels were; health workers, teachers, community leaders and religious leaders [4]. We expected the HPV vaccinated girls’ exposure to health information to have influenced their knowledge about HPV and HPV vaccine, their perceptions of sexual risk and their sexual behavior intentions. Our expectations on sexual risk perceptions and sexual behaviour intentions were premised in the Health Belief Model of health behavior (HBM). According to the HBM, individuals who perceive personal susceptibility to a serious health problem or its consequences are more likely to adopt preventive health behaviours [20–21]. We tested a hypothesis that; vaccinated girls were more knowledgeable about HPV and HPV vaccine, likely to perceive a higher sexual risk, and more likely to desire postponement sexual debut to avoid STIs after being exposed to information about HPV, a sexually transmitted infection.

## Methods

### Design

This comparative cross-sectional study was conducted between November and December 2011. Girls vaccinated against HPV were drawn from Ibanda district while unvaccinated participants for comparison were selected from Mbarara district.

### Participants

#### Selection of schools

Using multi-stage sampling, 3 rural sub-counties were randomly selected in Ibanda and 5 in Mbarara. Also, one urban sub-county was purposively selected in each district. Sixteen out of 120 schools were then randomly selected in Ibanda and 16 out of 66 schools in Mbarara, stratified by rural-urban location.

#### Study population and sample

In Ibanda, all assenting and consenting primary five and primary six girls present on the day of the survey who had received at least one dose of the HPV vaccine and whose parents or guardians gave written consent for their participation were recruited in the survey. Similar assent/consent criteria were followed for the unvaccinated comparison group in Mbarara. Using a formula for sample size calculation in studies comparing two proportions in terms of risk [22], a sample of 800 girls in P5 and P6 was targeted for the survey but only 777 girls (444 in Ibanda and 333 in Mbarara) participated. P6 study participants in Ibanda district had completed their vaccination 13 months before while their P5 colleagues completed vaccination one month prior to the date of data collection. Unvaccinated girls in Ibanda, school absentees and those who did not return the assent/consent forms in both districts were excluded. Data was analyzed for 670 who reported no sexual experience.

### Data collection

A pre-tested and translated self-administered questionnaire was used to collect data. Selected girls were assembled in a room on the day of the survey. Each girl filled the questionnaire under close supervision of the first author and research assistants who also explained to the girls the purpose and procedure of the survey. The questionnaire excluded respondents’ names to ensure their anonymity in the collected data. Data was collected first in Ibanda before Mbarara district.

### Variables and measurements

Independent variables were: HPV vaccination status, socio-demographic characteristics (age, kind of school [exclusively day/both day and boarding], location of school [rural/urban]), parental communication and perceived peer norms about delayed sexual debut. Dependent variables were: knowledge about HPV and HPV vaccine; perceived sexual risk, and intentions for sexual debut. Questions on HPV vaccination status and socio-demographic characteristics required yes/no answers. Each of the other variables was assessed through responses to a set of questions or statements, scored on a Likert scale. Scores for all questions in a variable were added to generate a subscale for the variable. The subscales were analyzed as dichotomous variables.

Parental communication was assessed by asking respondents how often their parents or guardians talked to them about their future and the dangers of pre-marital sex. Peer norms were assessed by asking each adolescent if most of her friends; would like to have sex at their current age, thought that one had to be older before having sex, thought she should not have sexual intercourse, and had been encouraging her to have sexual intercourse. Each adolescent was also asked if she believed sexual intercourse made a girl popular and if she believed sexual intercourse at her age was a “great” thing for a girl.

Knowledge was assessed through responses to 14 factual statements, some correct and others incorrect. These were mainly based on information about HPV and HPV vaccine contained in two leaflets, “Your guide to prevent cancer of the cervix” and “What you need to know about cancer of the cervix and its prevention”. The leaflets produced by the Ministry of Health and partners were used for sensitization prior to HPV vaccination. The statements were: HPV is the primary cause of cervical cancer, HPV is usually sexually transmitted, men cannot get the virus that causes cervical cancer, HPV can be transmitted through skin-to-skin genital contact without sexual intercourse, use of condoms during sex can completely prevent HPV, life-long sexual abstinence prevents HPV, and two doses of HPV vaccine can effectively protect a girl against HPV infection. Others were: HPV vaccine can cure individuals who are already infected with HPV, HPV vaccine can cause infertility, HPV vaccine protects against HIV, HPV vaccine protects against other STDs, young girls who are infected with HPV can develop cervical cancer, Uganda is the first country in the world to use the HPV vaccine, and it is possible for a healthy-looking person to have the HPV. Each statement had three answer choices (Yes, no and don’t know). The scale used to assess knowledge had good internal consistency (Cronbach’s α = .75).

Perceived sexual risk was assessed by questions on currently perceived chance of acquiring HIV and other sexually transmitted infections (STIs), separately. Each question had five answer choices (High, medium, low, uncertain and none). Scores for HIV and other STIs were combined during analysis; the resultant two-item sexual risk subscale had inadequate internal consistency (Cronbach’s α = .61). Intentions for sexual debut was assessed using a 12-item subscale adapted from a study by Khan et al (2004)[23]; it had good internal consistency (Cronbach’s α = .87).

### Statistical analysis

Data was analyzed using the Statistical Package for the Social Sciences (SPSS) version 16.0 [24]. Univariate analysis was conducted to generate frequencies and percentages for categorical variables. Means and standard deviations were generated for continuous variables. Independent samples t-test was used to compare continuous variables. Complex samples analysis was done by applying sampling weights to the statistics (means, percentages and odds ratios) to take care of the clustering effect of schools. At bivariate level, we evaluated relationships between independent variables (HPV vaccination status, socio-demographic characteristics, parental communication, and peer norms about sexual debut) and dependent variables (level of knowledge, perceived sexual risk and intentions for sexual debut). Pearson’s χ2 tests were used to determine statistical significance of differences observed. We performed complex samples logistic regression analysis to test the hypothesis that girls vaccinated against HPV were more knowledgeable, perceived higher sexual risk, and were more likely to support postponing sexual debut (PSD). In the analysis, we compared proportions of girls vaccinated against HPV with proportions of unvaccinated girls. Relevant Odds Ratios (ORs) and 95% Confidence Intervals (CI) determined the relationships between independent and dependant variables. Level of statistical significance was set at 0.05.

### Ethical approval

The research, “Human Papillomavirus (HPV) Vaccination and Adolescent Girls’ Knowledge and Sexuality in Western Uganda: A Comparative Cross-Sectional Study” was approved by the Higher Degrees Research and Ethics Committee of the then Makerere University Medical School (now College of Health Sciences) (REC REF 2011–200) and the Uganda National Council for Science and Technology (REF SS 2478). Additional approvals for this research were obtained from relevant local government and school authorities. Prior to the interviews in each selected school, parents or guardians gave written informed consent by filling and returning a form sent from school through their respective targeted children a day before the survey date. In addition, on the day of the survey written informed assent was obtained from the targeted minors (below 18 years of age) while older girls gave written informed consent.

## Results

### Respondents’ background characteristics

The overall mean age of girls was 13.3 years (SD = 1.39). The mean age of vaccinated girls (13.4 years, SD = 1.39) did not differ significantly with the mean age of unvaccinated girls (13.3 years, SD = 1.40). The results in Table 1 describe association of the adolescent girls’ background characteristics with their attitudes towards delayed sexual debut. High parental communication was significantly associated with attitudes that supported PSD (Crude OR: 1.92, CI: 1.13–3.26; *p* = 0.02).

**Table 1:** Background characteristics of respondents by attitudes towards postponement of sexual debut (PSD).

	Attitudes towards PSD (N = 646)		
Background characteristics of respondents	Likely to postpone sexual debut (n = 357) *(%)*	Unlikely to postpone sexual debut (n = 289) *(%)*	*p* value
**HPV vaccination status** ^**1**^			
Vaccinated	208 (71.7)	162 (69.9)	0. 68
Unvaccinated	149 (28.3)	127 (30.1)	1.0
**Age**			
Younger adolescent (9–14 years)	286 (78.9)	237 (82.2)	0.25
Older adolescent (15–19 years)	71 (21.1)	52 (17.8)	1.0
**Kind of school**			
Exclusively day school	269 (73.8)	229 (76.8)	0.51
Both day and boarding school	88 (26.2)	60 (23.2)	1.0
**Location of school**			
Rural	234 (66.1)	202 (72.8)	0.20
Urban	123 (33.9)	87 (27.2)	1.0
**Parental communication**			
High parental communication	312 (88.5)	228 (80.1)	0.02
Low parental communication	45 (11.5)	61 (19.9)	1.0
**Peer norms about delayed sexual debut**			
Norms support delayed sexual debut	315 (88.8)^+^	262 (90.1)	0.61
Norms do not support delayed sexual debut	41 (11.2)	27 (9.9)	1.0

**Notes**:

^1^ Vaccinated represents the sample from Ibanda; unvaccinated represents the Mbarara sample.

^+^Figures do not add up to 357 due to a missing case.

Background characteristics are the predictors while attitude towards postponement of sexual debut is the outcome.

For all background or predictor variables, 2^nd^ column odds are divided by 3^rd^ column odds.

Significance is at *p* ≤ 0.05.

### Adolescent girls’ knowledge about HPV and HPV vaccine

Knowledge was low; 102/670 or 17.6% of the adolescents overall were knowledgeable. A bivariate analysis to identify association of the girls’ background characteristics with their knowledge showed that significantly more vaccinated girls (87/385 or 22.6%) than unvaccinated (15/285 or 5.3%) were knowledgeable (Crude OR: 5.26, CI: 2.32–11.93; *p* = 0.000).

### Adolescent girls’ perceptions of sexual risk

Perception of sexual risk (risk for HIV and other STIs infection) was low; 17.9% of the girls overall perceived a high sexual risk. Table 2 describes association of the adolescent girls’ background characteristics with their perceptions of sexual risk. Being vaccinated against HPV was significantly associated with perception of a high sexual risk (Crude OR: 1.60, CI: 1.03–2.48; *p* = 0.04). Being a younger adolescent was significantly associated with perception of a low sexual risk (Crude OR: 0.45, CI: 0.26–0.80; *p* = 0.007). Knowledge was significantly associated with perception of a high sexual risk (Crude OR: 3.41, CI: 1.60–7.24; *p* = 0.002).

**Table 2:** Background characteristics of respondents by level of perceived sexual risk.

	Level of perceived sexual risk (N = 670)		
Background characteristics of respondents	High risk (n = 114) *(%)*	Low risk (n = 556) *(%)*	*p* value
**HPV vaccination status** ^**1**^			
Vaccinated	76 (78.4)	309 (69.5)	0. 04
Unvaccinated	38 (21.6)	247 (30.5)	
**Age**			
Younger adolescent (9–14 years)	81 (68.7)	461 (82.9)	0.01
Older adolescent (15–19 years)	33 (31.3)	95 (17.1)	
**Kind of school**			
Exclusively day school	92 (78.7)	423 (73.9)	0.31
Both day and boarding school	22 (21.3)	133 (26.1)	
**Location of school**			
Rural	83 (73.1)	370 (68.2)	0.42
Urban	31 (26.9)	186 (31.8)	
**Parental communication**			
High parental communication	91 (80.9)	470 (85.7)	0.34
Low parental communication	23 (19.1)	86 (14.3)	
**Knowledge of cervical cancer and HPV vaccine**			
Knowledgeable	35 (35.2)	67 (13.7)	0.01
Not knowledgeable	79 (64.8)	489 (86.3)	

**Notes**:

^1^ Vaccinated represents the sample from Ibanda; unvaccinated represents the Mbarara sample.

Background characteristics are the predictors while perceived risk for HIV and other STIs infection is the outcome.

For all background or predictor variables, high risk odds are divided by low risk odds.

Significance is at *p* ≤ 0.05.

Logistic regression analysis was conducted with perceived sexual risk as the dependent variable. Predictor variables were the significant socio-demographic variables in bivariate analysis, HPV vaccination status, and knowledge. As shown in Table 3, HPV vaccination did not predict perception of a high sexual risk after controlling for other background variables. Being a younger adolescent predicted perception of a low sexual risk even after controlling for other background variables (*p* = 0.008). Knowledge was significantly associated with perception of a high sexual risk even after controlling for other background variables (*p* = 0.008).

**Table 3:** Predictors of perceived sexual risk derived by logistic regression analysis.

Respondents’ characteristics	Perceived sexual risk (N = 670) [Adjusted ORs (95% CI)]
Vaccinated against HPV	1.22 (0.74–2.02)
Younger adolescent (9–14 years)	0.49 (0.29–0.82)**
Knowledgeable about HPV and HPV vaccine	3.12 (1.37–7.13)**

**Note**:

** Significant at *p* ≤ 0.01

### Adolescents’ intentions for sexual debut

We expected that being vaccinated against HPV, being knowledgeable and perceiving a high sexual risk would be associated with adolescents’ support for PSD. Consistent with expectation, vaccinated girls (208/370 or 56.2%) were more likely to support delayed sexual debut compared to the unvaccinated (149/276 or 54.0%) but the difference was not statistically significant (Crude OR: 1.09, CI: 0.70–1.71; *p* = 0.68). As expected, knowledgeable girls were more likely to support delayed sexual debut (60/100 or 61.0%) than the unknowledgeable (297/546 or 54.4%) although the difference was not statistically significant (Crude OR: 1.31, CI: 0.81–2.12; *p* = 0.26). Girls who perceived a high sexual risk were more likely to support delayed sexual debut (65/111 or 59.8%) than those who perceived a low risk (292/535 or 54.6%) but the difference was not significant (Crude OR: 1.24, CI: 0.75–2.04; *p* = 0.39).

Variables entered in the model of logistic regression to predict attitudes/beliefs about PSD were; parental communication that was significantly associated with intentions for sexual debut in bivariate analysis and the study hypothesized predictors: HPV vaccination, knowledge, and perceived sexual risk. The results are presented in Table 4. HPV vaccination did not predict intentions for sexual debut even after controlling for other background variables, knowledge and sexual risk perception. Also, knowledge and perception of a high sexual risk did not significantly predict intentions for sexual debut. High parental communication predicted intentions to delay sexual debut even after controlling for vaccination status and the other study hypothesized predictors (Adjusted OR: 1.93, CI: 1.13–3.39; *p* = 0.02).

**Table 4:** Predictors of attitudes towards postponement of sexual debut (PSD) derived by logistic regression analysis.

Respondents’ characteristics	Adjusted ORs (95% CI)
Vaccinated against HPV	0.99 (0.63–1.56)
High parental communication	1.95 (1.13–3.39)*
Knowledgeable about HPV and HPV vaccine	1.21 (0.77–1.89)
Perceiving high risk for infection with HIV and other STIs	1.04 (0.59–1.83)

**Notes:** Number of observations = 645.

*Significant at *p* ≤ 0.05.

## Discussion

It was anticipated that HPV-vaccinated girls were more knowledgeable, perceived a higher sexual risk, and were more likely to support delayed sexual debut owing to their exposure to health information during the HPV vaccination. This was premised on a hypothesis that vaccination against an STI could provide an opportunity to increase the girls’ awareness about their sexual risk when they become sexually active, and the need for prevention [13, 19].

As expected in this study, HPV vaccination was associated with being knowledgeable. Notably, although significantly more vaccinated than unvaccinated girls were knowledgeable it is a minority (23%) of vaccinated girls that were knowledgeable. Low knowledge has been documented in other post-vaccination studies [25–26]. However, knowledge was lower in our study compared to other studies. The 23% in our study was lower than the overall knowledge level in the study in England [26] where 45% of vaccinated school girls had high knowledge about HPV and HPV vaccine. Like in Uganda, information was provided to school girls in England through printed materials. Concerning knowledge of specific aspects of HPV and HPV vaccine: 51% of vaccinated girls in our study compared to 61% of vaccinated adolescents and young women in another study [27] answered correctly that HPV is mainly sexually transmitted; 32% compared to 86% [27] answered correctly that HPV vaccine does not protect against all STDs; 35% compared to 55% in another study [26] answered correctly that HPV can be transmitted through skin-to-skin genital contact without sexual intercourse; 40% compared to 70% [26] knew that HPV vaccine requires 3 doses; 24% compared to 28% [26] knew that men can get HPV; and 22% compared to 53% [26] and 51% [25] answered correctly that HPV causes cervical cancer. Low knowledge in our study implies that the information that was disseminated through leaflets prior to vaccination against HPV did not greatly impact the girls’ knowledge. It suggests a necessity to re-consider the use of printed materials during sensitization prior to vaccination. A study in England [28] doubted sufficiency of written information in communicating HPV, cervical cancer and HPV vaccine information to some adolescents and noted that additional non-written information sources, such as discussions with youth workers, may be beneficial.

In this study, vaccination against HPV was associated with knowledge and knowledge predicted perception of a high sexual risk. Contrary to expectation however, HPV vaccination did not predict perception of sexual risk. This could be attributed to the low level of knowledge even among vaccinated girls. Previous studies of risk perception after HPV vaccination reported that higher knowledge was associated with a higher awareness of the risk of sexual contact [29] and perceived need for safer sexual behaviours [30–31]. Low knowledge on the other hand was associated with high “personal compensation” beliefs whereby girls indicated that they would be more likely to have sex and more likely to have unprotected sex if they had the HPV vaccination [28], suggesting they would feel less at risk of infection with HPV and other STIs after vaccination. In particular, lack of knowledge about the sexual transmissibility of HPV would imply less opportunity for recipients of the HPV vaccine to learn to practice safe sexual behaviours. This would increase the girls’ risk of infection with other STIs and other HPV sub-types not prevented by the currently available HPV vaccines. Future vaccination programs should have sound provisions for maximizing adolescents’ awareness of HPV and other STIs as pertinent risks of sexual contact so as to enable the girls appreciate the need for adoption of safer sexual behaviours even after getting the HPV vaccine.

We expected HPV-vaccinated girls to be more likely to intend to delay sexual debut, but results showed that HPV vaccination, knowledge, and perceived sexual risk did not predict intentions for sexual debut. This was consistent with previous studies that showed no association of HPV vaccination with adolescent and young women’s sexual initiation and activity [18, 32–34]. The finding supported the pro-HPV-vaccine argument, which holds that the vaccine has a negligible impact on adolescents’ sexual behaviour because initiation of sexual activity is influenced by multiple factors [35]. The finding contradicted the fairly widely reported fear among HPV-vaccine critics that vaccination against an STI could lead to adolescent girls’ sexual activity [4, 8, 13–14]. The apprehension about possible influence of HPV vaccination on adolescent sexual behaviour is largely grounded in the road-safety-based theory of risk compensation, which holds that people always adjust their behaviour to attain a balance between the amount of risk they perceive and their target level of risk [36]. In the HPV context, the idea of risk compensation in terms of sexual behavior assumes that immunized girls would believe that HPV vaccine protects against STIs generally rather than only cervical cancer [28], which would likely lead to their early sexual activity. However, results of this study revealed that perception of sexual risk did not influence adolescents’ intentions for sexual debut. This was largely in agreement with a study, which reported that fear of STIs was not a major motivation for abstinence among 15–19-year-olds who had never had sex [37]. Also unexpectedly, this study found that knowledge did not predict intentions for sexual debut. This finding is supported by results of several sexual-behaviour studies in Uganda showing that awareness of sexual risk did not necessarily translate into positive sexual behavioural changes [38–41]. Although our results largely dispel the fear that vaccination against HPV could encourage adolescents’ sexual activity, it remains unclear what the effect would be if the adolescents in the study were more knowledgeable. Nonetheless, HPV vaccine sensitization programs should aim to maximize adolescents’ and their parents’ knowledge because safe sexual behaviours remain important even for those who are vaccinated against HPV. Low knowledge among parents and adolescents has been associated with adolescent perception of less need for safer sexual behaviors after HPV vaccination [31].

In this study, high parental communication irrespective of vaccination status predicted adolescent attitudes that were likely to support PSD. This was consistent with previous adolescent studies, not necessarily related to HPV vaccination, which reported PSD to be positively associated with parental communication [42–43]. Our concept of parental communication covered parent-adolescent discussion about the adolescents’ future and about the dangers of premarital sex. This suggests that adoption of more effective communication approaches and promoting parent-adolescent communication about risks of sexual contact during HPV vaccine sensitization could promote adolescent attitudes that support PSD.

This study has some methodological limitations to be considered while interpreting the findings. The research design was cross-sectional, rendering it difficult to make definitive inferences about the direction of the observed relationships between dependent and independent variables. Adolescents’ self-reported perceptions of sexual risk and sexual behaviour intentions may change with time. The vaccinated P6 study participants had completed their vaccination about one year before while their P5 colleagues completed vaccination about one month prior to the date of data collection. It is therefore possible that long term socio-behavioural effects of the vaccination were yet to be evident. Since the P6 vaccinated girls were interviewed about one year after their vaccination, some of their responses could have been compromised due to recall bias. There was also a risk of contamination arising from comparing bordering districts. This and the risk of recall bias were minimised by limiting our assessment of knowledge to the information in leaflets that were distributed in the targeted schools. In all, the sample size was relatively large, thereby increasing the power of the study. Data that was collected and its subsequent interpretation are reasonably valid and reliable for programming and policy purposes.

### Implications and contribution

Levels of knowledge and perceived sexual risk were low among vaccinated girls despite the pre-vaccine sensitization. Yet, knowledge predicted perception of a high sexual risk. Sensitization strategies should be revised to increase their effectiveness, improve adolescents’ awareness of HPV sub-types not prevented by the currently available vaccines, HIV and other STIs as pertinent risks of sexual contact. This would likely pave the way for adoption of safer sexual behaviours which remain necessary even after individuals have been vaccinated against HPV. Our results showing parental communication as a steady predictor of attitudes that support PSD suggest that involvement of parents and promotion of parental communication would likely enhance effectiveness of strategies to promote safer sexual behaviours of adolescents after vaccination. HPV vaccination, knowledge and perceptions of sexual risk did not predict adolescent girls’ intentions for sexual debut. Therefore, our study adds to the body of evidence that vaccination against HPV is unlikely to encourage adolescent sexual activity.

### Conclusion

HPV vaccination was associated with being knowledgeable. HPV vaccination did not influence perception of sexual risk but knowledge did. HPV vaccination, knowledge, and sexual risk perception did not influence sexual behaviour intentions. This implies that HPV vaccination is not likely to encourage adolescent sexual activity. However, this study did not decisively explain the influence of knowledge on adolescents’ sexual behaviour intentions due to the low levels of knowledge even among vaccinated girls. Further prospective cohort studies are needed to get more definitive results on the possible behavioral effects of HPV vaccination and its accompanying knowledge. Future research would be more helpful if it could be interventional.
